# Newborn Screening for Congenital Hypothyroidism

**DOI:** 10.4274/Jcrpe.845

**Published:** 2013-03-01

**Authors:** Atilla Büyükgebiz

**Affiliations:** 1 İstanbul Bilim University, Deparment of Pediatric Endocrinology, İstanbul, Turkey

**Keywords:** Neonatal screening, congenital hypothyroidism, iodine deficiency

## Abstract

Newborn screening (NS) for congenital hypothyroidism (CH) is one of the major achievements in preventive medicine. Most neonates born with CH have normal appearance and no detectable physical signs. Hypothyroidism in the newborn period is almost always overlooked, and delayed diagnosis leads to the most severe outcome of CH, mental retardation, emphasizing the importance of NS. Blood spot thyroid stimulating hormone (TSH) or thyroxine (T4) or both can be used for CH screening. The latter is more sensitive but not cost-effective, so screening by TSH or T4 is used in different programs around the world. TSH screening was shown to be more specific in the diagnosis of CH. T4 screening is more sensitive in detecting especially those newborns with rare hypothalamic-pituitary-hypothyroidism, but it is less specific with a high frequency of false positives mainly in low birth weight and premature infants. The time at which the sample is taken may vary. In the majority of the centers, blood is obtained from a heel prick after 24 hours of age to minimize the false positive high TSH due to the physiological neonatal TSH surge that elevates TSH levels and causes dynamic T4 and T3 changes in the first 1 or 2 days after birth. Early discharge of mothers postpartum has increased the ratio of false positive TSH elevations. Although transient hypothyroidism may occur frequently, all these infants should be treated as having CH for the first 3 years of life, taking into account the risk of mental retardation. A reevaluation after 3 years is needed in such patients. The goal of initial therapy in CH is to minimize neonatal central nervous system exposure to hypothyroidism by normalizing thyroid function, as rapidly as possible.

**Conflict of interest:**None declared.

## INTRODUCTION

Congenital hypothyroidism (CH) is the commonest treatable cause of mental retardation. It is one of the most common disorders related to mental impairment and growth retardation in newborns. In many countries, neonatal thyroid screening programs are performed for early diagnosis and treatment of hypothyroidism. CH is usually sporadic and occurs in one in 3000-4000 infants. Most infants with CH are normal at birth and show no signs, emphasizing the importance of screening programs in early detection of CH (1,2).

Newborn screening (NS) for CH is one of the major achievements of preventive medicine. Although since 1972 the problem of CH has been resolved in developed countries by the implementation of NS, the same cannot be said for developing countries that still have no NS programs for CH (2,3). Since diagnosis based on clinical findings is delayed in most instances because of few symptoms and signs, hypothyroidism in the newborn period is almost always overlooked, and delayed diagnosis leads to the most severe outcome of CH, namely, mental retardation. In a Danish study (4) conducted on infants born between 1970 and 1975, it was emphasized that only 10% of the affected infants were diagnosed within the first month of life, 35% within 3 months, and 70% within the first year. In the remainder of the infants, the diagnosis was delayed to the 3^rd^ and 4^th^ years of life. In a retrospective analysis of 1000 cases of CH from Turkey (5), the mean age at diagnosis was reported to be 49 months, and only 3.1% of cases were diagnosed within the first month, while 55.4% were diagnosed after 2 years of age.

The first CH screening was performed by Dussault (6,7), in Quebec-Canada in 1972. They detected 7 hypothyroid infants among 47 000 newborns during a 3-year period. The high frequency of false positives delayed the diagnosis and increased the cost, and they therefore devised cut-offs values to be used for recall. Thyroid hormone and thyroid-stimulating hormone (TSH) levels were assessed in the recalled group of babies. In the meantime, radioactively labeled antibodies for determining thyroxine (T4) in dried blood spots were introduced regionally in the USA and in Europe. Screening programs for CH went parallel with screening programs of phenylketonuria. In the initial report by Dussault et al (8), the method was recommended as a confirmatory test, knowing that it would miss cases with hypothalamic-pituitary hypothyroidism, which they reported to constitute 10% of the cases. In 1976, Walfish (9) reported in the Lancet that cord blood TSH measurements had greater sensitivity and specificity as compared to cord blood T4 and spot blood (collected on 3 to 4 day old newborns) T4 results and that both false positives and costs were higher in the T4 method. This same author also suggested routine T4 supplemented by TSH estimation be used in mass screening. Although more sensitive, screening by T4 and TSH together is not cost-effective, therefore, mostly TSH, and rarely T4 screening, is used around the world. In Europe, TSH screening is preferred, whilst some centers in the USA prefer primary T4 testing supplemented by TSH (10,11,12,1,3,14). The recall rate for primary hypothyroidism in both approaches is 0.05%, and the rate of false positive results is higher using primary T4 strategy. TSH screening was shown to be more specific in the diagnosis of CH, while T4 screening was more sensitive in detecting newborns with rare hypothalamic-pituitary hypothyroidism but less specific with a high frequency of false positives mainly in low birth weight and premature babies. T4-binding globulin (TBG)-deficient babies who are euthyroid and who were not targets for NS, could also be detected by T4 screening. The Neonatal Thyroid Screening Conference held in Tokyo in 1982 recommended NS programs oriented to detect infants with elevated serum concentrations of TSH. It has been also suggested that this could be accomplished by measuring TSH in filter paper blood spot or by measuring T4 supplemented by TSH on the same blood spot of infants who have T4 values in the lower 3^rd^ to 10^th^ percentile (15).

**Methods**

The aim of NS programs is to detect all cases with the disease as early as possible, with an acceptable cost-benefit ratio and to avoid false positive results. In recent years, more sensitive and automated methods (chemiluminescence, fluoroimmunoassay, etc.) for determining both TSH and T4 in dried blood spots have been introduced (16,17,18,19,20,21). These new methods have increased sensitivity and specificity in the detection of CH. However, despite the development of more accurate test programs, approximately 5% of CH cases may still be missed in any screening program. The reasons could be failure of sample collection, unsatisfactory samples, misinterpretation of samples and unsatisfactory recalls, the condition being subclinical or, as is true for programs which measure only TSH, failure to detect infants with central CH (14,15,16,17).

The ideal time to obtain the blood spot is 3-5 days after birth to minimize the false positive high TSH values due to the physiological neonatal TSH surge that elevates TSH levels and causes dynamic T4 and total triiodothyronine (T3) changes in the first 1 or 2 days after birth. Early discharge of mothers postpartum has increased the ratio of false positive TSH elevations from 3:1 to 5:1. The difficulty in screening for CH using cord blood samples is with the handling and transporting the samples, making it an impractical method for mass screening (22).

In some laboratories, the threshold cut-off is adjusted based on the age of the infant when the blood spot is obtained. The cutoff for reporting an elevated TSH is a level above 20 to 25 mU/mL in most screening programs. Whichever method is used, babies whose initial TSH is >50 mU/mL are most likely to have permanent CH, whereas a TSH level between 20 and 49 mU/mL is frequently a false positive or represents transient hypothyroidism. Transient CH is particularly common in premature infants in borderline iodine-deficient areas. 

In the primary TSH method (immunofluorometric method), when 15 mU/mL is used as cutoff, the recall rate is quite low (0.05%). Iodine deficiency could increase false positives and increase recall rate up to 3% .The sensitivity of the TSH method for CH is reported to be 97.5% and its specificity is 99% (2,23,24). CH is reportedly increasing in the United States, possibly reflecting changes in screening methods. The TSH cutoff value has been decreased from 15 mU/mL to 5 mU/mL, thus adding cases that have predominantly functional disorders which impact on intellectual disability, if left untreated, remains to be determined (24).

In short, NS with primary TSH method detects overt and compensated primary hypothyroidism but misses secondary/ tertiary hypothyroidism, TBG deficiency, and premature infants with very low body weight with delayed TSH surge.In primary T4 screening, performed in some states of the USA, cutoff at the 10^th^ percentile of T4 values resulted in 1.5% missed cases, whereas cutoff at the 5^th^ percentile resulted in 3.5% missed cases. Only 2% of cases were missed using the 20th percentile as a cutoff, but of course, with increased cost in terms of repeat testing (25). Optimal screening requires initial T4 determination to be followed by TSH determinations in case of low T4 samples. In short, NS with primary T4 method detects overt primary hypothyroidism, secondary/tertiary hypothyroidism (1 in 50 000-100 000 live births), hypothyroxinemia in sick and preterm newborns, TBG deficiency, and hyperthyroxinemia but misses compensatory hypothyroidism with subnormal T4 and elevated TSH levels as well as transient hyperthyrotropinemia when iodine deficiency is present.

The reliability of the laboratories is as crucial as the reliability of the detection methods (with emphasis on sensitivity, specificity, and positive predictive value). According to the recommendations of the Working Group of Neonatal Screening of European Society for Pediatric Endocrinology (ESPE), screening should be conducted in centralized laboratories covering 100 000 newborns per year (26,27,28). These laboratories should participate in international control programs. In North America, it is estimated that 6-12% of the neonates with CH are missed due to biological factors and screening errors (27,28).

**Results and Discussion**

**Hypothyroxinemia(low T4 and normal TSH)**

This condition occurs most commonly in premature infants and is found in 50% of babies born less than 30 weeks of gestation (26). Screening programs that employ primary TSH analysis will miss these infants because of normal TSH levels. Often, the free T4 (fT4) is less affected than the total T4. The reasons for the hypothyroxinemia of prematurity are complex. In addition to hypothalamo-pituitary immaturity, low TBG levels and decreased conversion of T4 to T3 exists in premature babies. Numerous studies have shown that there is a correlation between the degree of lowering of T4 and negative outcomes such as mortality and development problems. Systematic supplementation of all low birth weight babies is not recommended at this time (23,29,30).

Other causes of low T4 in the face of normal TSH are the euthyroid sick syndrome, TBG deficiency, laboratory errors, and central hypothyroidism (3). Immature liver function, undernutrition, and illness are the reasons for low T4 and normal TSH levels in euthyroid sick syndrome. Euthyroid sick syndrome may be seen in the sick term newborns as well (23). TBG deficiency is an X-linked condition discovered only by screening programs using the primary T4 approach. It does not require treatment since the plasma levels of free thyroid hormones are normal, and the subjects are euthyroid. Its incidence is estimated to be 1 in 2800 (31). TBG deficiency should be investigated especially in male infants with low T4 and normal TSH and could be confirmed by measuring TBG levels in the serum. Loss of protein from nephrotic syndrome may also lead to low total T4. Errors in measurement may be caused by errors in sample gathering, impregnation with water due to improper sample handling or extreme hematocrit values which adversely affect the measurements.

In a term neonate with a low fT4 but normal TSH level, true central hypothyroidism, which is quite rare, should be ruled out. Mutations in the gene coding for the beta subunit of TSH or the thyrotropin releasing hormone receptor could be the causes (32,33). Central hypothyroidism could coincide with other anterior pituitary hormone deficiencies; hypoglycemia, microphallus, prolonged jaundice and/or cryptorchidism (34,35,36).

**Isolated Hyperthyrotropinemia (Normal T4 and Elevated TSH)**

Elevated TSH, despite a normal or low T4, indicates inadequate hormone production. It is most common in premature babies. Although some babies have compensated hypothyroidism, the etiology is not clear in the others. In early discharged babies (in the first day or two), because of the cold-induced TSH surge, TSH values are found to be elevated. It could be a transient finding due to goitrogens, iodine deficiency, or medications. Genetic defects of hormone biosynthesis and also dysgenesis, especially ectopia, could be the causes. Elevated TSH levels with normal T4 levels could persist for years. Iodine excess, especially when iodine-containing antiseptics are used, may cause transient hypothyroxinemia in preterm babies (37,38).

**Low T4 and Elevated TSH**

Primary CH is the most common cause of this condition. However, transient cases, which may be caused by maternal antithyroid medication, exposure to topical iodine, maternal iodine deficiency or excess, maternal TSH receptor blocking antibodies, medications (dopamines, steroids), or prematurity (<30 weeks), may also occur and are not rare. All these cases should be treated as CH for the first 3 years of life by taking into account the risks of mental retardation. A re-evaluation after age 3 years is needed in such patients (1,38,39,40).

**Conclusion**

The outcomes of early diagnosis and treatment of CH are remarkable because in these patients, the average intelligence quotient values at 7 years are in the normal range (41). The scientific gains in laboratory research have enabled clinicians to improve the lives of children with CH. Development of sensitive assays to measure serum T4 and TSH using a blood spot made it possible to initiate newborn thyroid screening programs. Early diagnosis and treatment with adequate doses of L-T4 have rescued affected children from a life of mental retardation. Before 1972, case detection was the only method of diagnosis. Unfortunately, the majority of CH babies in that era were permanently neurologically injured when treatment with beef thyroid extracts was initiated at 3 or more months of age. Today, NS enables diagnosis and treatment which can usually be accomplished within 2 weeks after birth. NS experience has shown that the biochemical evidence of CH is present long before the physical signs appear. 

Today, NS for CH is accepted as a tool in the context of primary health care for infants like breast feeding, immunization and oral rehydration. Our aim as scientists working in either the developed or the developing world should be to help establish NS programs in countries that have no national NS programs.
